# Pharmacological modulation of chloride channels as a therapeutic strategy for neurological disorders

**DOI:** 10.3389/fphys.2023.1122444

**Published:** 2023-03-02

**Authors:** Zhiyu Wang, Kaylee Choi

**Affiliations:** Lead Discovery & Characterization, Therapeutic Discovery, Amgen Research, South San Francisco, CA, United States

**Keywords:** chloride homeostasis, chloride channels, central nervous system, small molecules, neurological disorders

## Abstract

Chloride homeostasis is critical in the physiological functions of the central nervous system (CNS). Its concentration is precisely regulated by multiple ion-transporting proteins such as chloride channels and transporters that are widely distributed in the brain cells, including neurons and glia. Unlike ion transporters, chloride channels provide rapid responses to efficiently regulate ion flux. Some of chloride channels are also permeable to selected organic anions such as glutamate and γ-aminobutyric acid, suggesting neuroexcitatory and neuroinhibitory functions while gating. Dysregulated chloride channels are implicated in neurological disorders, e.g., ischemia and neuroinflammation. Modulation of chloride homeostasis through chloride channels has been suggested as a potential therapeutic approach for neurological disorders. The drug design for CNS diseases is challenging because it requires the therapeutics to traverse the blood-brain-barrier. Small molecules are a well-established modality with better cell permeability due to their lower molecular weight and flexibility for structure optimization compared to biologics. In this article, we describe the important roles of chloride homeostasis in each type of brain cells and introduce selected chloride channels identified in the CNS. We then discuss the contribution of their dysregulations towards the pathogenesis of neurological disorders, emphasizing the potential of targeting chloride channels as a therapeutic strategy for CNS disease treatment. Along with this literature survey, we summarize the small molecules that modulate chloride channels and propose the potential strategy of optimizing existing drugs to brain-penetrants to support future CNS drug discovery.

## 1 Introduction

As the most abundant anion, chloride performs multiple physiological functions in the cells, where it maintains cellular homeostasis (Verkman and Galietta, 2021). In the central nervous system (CNS), chloride participates in multiple events to support neuronal functions. For instance, chloride regulates postsynaptic inhibition involved in neural coding through GABA_A_ receptors (GABA_A_Rs; GABA, γ-aminobutyric acid) (Doyon et al., 2016). Its concentration gradient directly impacts neuronal excitation and inhibition (Mahadevan et al., 2014). In addition, chloride mediates the physiological properties of the CNS-supporting cells, glia. Glial cells comprise astrocytes, microglia, and oligodendrocytes. *Astrocytes* are the most abundant glial cells in the brain, where they surround neurons to provide physical structures, maintain ion balances, regulate a blood flow, participate in neural repairs, and release and uptake neurotransmitters. These functions are regulated by chloride in multiple dimensions. For instance, the strength of Cl^−^ current is associated with the activity of glutamate transporters in astrocytes (Wilson and Mongin, 2019). Under transient ischemic stress, chloride is maintained at a dynamic balance through multiple chloride-transporting mechanisms to prevent astrocytic swelling (Engels et al., 2021). *Microglia*, ‘brain macrophages’, keep sensing the microenvironment with a ramified morphology at the resting stage. Upon the recognition of foreign invaders such as pathogens or inflammatory molecules, they are activated to initiate immune responses with an alternation into amoeboid morphology initiating neuroinflammation. This activation with a consequent morphology change is tightly regulated by chloride. Chloride, to be specific, participates in the membrane stretch during ramification of microglia and the associated tyrosine-phosphorylation signaling pathway (Eder et al., 1998). Its influx also provokes lamellipodium formation, suggesting the critical role in microglia migration towards foreign species (Zierler et al., 2008). Upon activation, chloride mediates phagocytosis and the release of proinflammatory cytokines from microglia, suggesting the therapeutic potential of chloride channel modulators for microglia-involved neurodegenerative diseases (Schlichter et al., 1996; Eder et al., 1998; Novarino et al., 2004; Zierler et al., 2008). *Oligodendrocytes* are the myelinating glia in the CNS. They assemble myelin sheath along nerve cell axons, reducing internodal membrane capacitance and facilitating rapid conduction of electrical impulses (Stassart et al., 2018). Its proliferation, development, and maturation require chloride homeostasis (Magalhães and Rivera, 2016).

Chloride homeostasis is regulated by multiple chloride-transporting proteins including ion channels and transporters. Due to genetic disorders, acute injuries, or inflammation, however, these functional proteins are dysregulated, contributing towards the pathophysiology of numerous neurological disorders such as epilepsy, autism, ataxia, hyperekplexia, and neuropathic pain (Funk et al., 2008; Kahle et al., 2008; Tyzio et al., 2014; Wu et al., 2016; Wu et al., 2022). Modulation of chloride homeostasis in the CNS has been suggested as a promising therapeutic approach to resolve chloride disturbance and associated pathological disorders.

The drug design for CNS diseases is challenging because it requires the therapeutics to traverse the blood-brain-barrier (BBB). Compared to biologics, small molecules exhibit better permeability to BBB and cellular membranes and offer flexibility for hit discovery and lead optimization. In the past years, tremendous efforts have been made to modulate chloride homeostasis through chloride transporting proteins. Unlike the transporters, chloride channels provide fast responses to efficiently regulate ion flux driven by electrochemical gradient. In addition, various chloride channels are permeable to larger anions such as GABA and glutamate, exhibiting neuroinhibitory and neuroexcitatory effects while gating (Park et al., 2009; Lee et al., 2010; Woo et al., 2012). These features suggest a promising strategy to regulate chloride-involved neurological disorders through chloride channels.

Since chloride channels show distinct properties between the CNS and peripheral systems (Zhang et al., 2004), herein, we describe the important roles of chloride homeostasis in each type of brain cells and introduce selected chloride channels in the CNS, emphasizing the contributions of their dysregulations towards the pathogenesis of CNS disorders. We also discuss targeting chloride channels as a therapeutic strategy for CNS disease treatments and review the small molecules that modulate chloride homeostasis and associated neurological disorders. From medicinal chemistry perspective, we calculate the physicochemical properties of these molecules and propose potential strategies to optimize specific physicochemical parameters through structural modification, supporting future CNS drug discovery.

## 2 Chloride channels in the CNS

### 2.1 Voltage-gated chloride channel (ClC family)

ClC channels are expressed on plasma membranes, intracellular organelles, and vesicles, where they regulate chloride gradients for various cellular functions. For instance, ClC-2 regulates intracellular chloride concentration of hippocampal pyramidal neurons through chloride extrusion based on its inward rectifying property (Rinke et al., 2010; Földy et al., 2010). The gating mechanisms of ClCs have been reported with their protein structures (Accardi, 2015; Poroca et al., 2017). Briefly, the subunit of dimeric ClC channels harbors an ion pore that is modulated by the protonation-deprotonation cycle of a glutamate gate. This cycle is voltage-dependent and can be initiated by repulsion or protonation when voltage navigates a permeant anion or a proton in. ClC family comprises nine members that can be divided into chloride channels (ClC-1, -2, -Ka, and -Kb) and Cl^−^/H^+^ exchangers (ClC-3 through -7). The contributions of ClCs in physiology and disease progressions have been extensively reviewed by Jentsch and colleagues (Jentsch and Pusch, 2018). In this section, we focus on the roles of ClC-1 and -2 in neurodegenerative diseases.

#### 2.1.1 ClC-1

In the CNS, ClC-1 is distributed in the hippocampus, brain stem nuclei, thalamic nuclei, and frontal neocortex, participating in physiological processes (Chen et al., 2013). ClC-1 in the CNS exhibits features distinct from ClC-1 in muscle tissue. For example, ClC-1 in astrocytes exhibits less dependence on voltage and extracellular Cl^−^ than that in skeletal muscle (Zhang et al., 2004). In the CNS, ClC-1 contributes to neuronal network maturation and neuronal excitability, suggesting its important role in preventing neurological disorders such as epilepsy (Rahmati et al., 2018). Furthermore, a parallel exome sequencing of 237 ion channel genes verifies that ClC-1 is involved in the pathogenesis of epilepsy (Chen et al., 2013).

Several drugs targeting ClC-1 have been developed to treat neuromuscular diseases. Acetazolamide influences the voltage-dependent gating of ClC-1, elevating open probability and chloride conductance (Eguchi et al., 2006). Acetazolamide, however, contains a primary sulfonamide group (see Table 1), which might impact the permeability across BBB (Wang et al., 2021). NMD Pharma, a clinical-stage biotech company, developed NMD670, a small-molecule inhibitor against ClC-1, recently granted Orphan Drug Designation by the Food and Drug Administration (FDA) for the treatment of myasthenia gravis. Myasthenia gravis is caused by autoimmunity against nicotinic acetylcholine receptors in skeletal muscle endplates in most cases (Koneczny and HerbstGravis, 2019). Therefore, the expected effect of NMD670 would be due to increased muscle excitability by blocking muscle ClC-1.

**TABLE 1:** Summary of chloride channels involved in neurological disorders and their small-molecule modulators with physiochemical properties.

Chloride channels	Gating mechanism	Related neurological disorders	Small-molecule modulators	*In vitro* potency	Development stage	Physicochemical properties			
						HBD (<3)	cLogP (2.0–4.0)	PSA (<90)	MW (<450)
ClC-1	Voltage-gated	Epilepsy	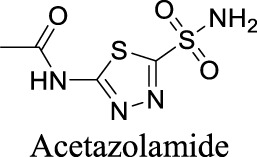	N/A	Approved drug for the treatments of glaucoma, epilepsy, altitude sickness, periodic paralysis, idiopathic intracranial hypertension, urine alkalinization, and heart failure	2	−0.98	114	222
ClC-2		Epilepsy; MLC	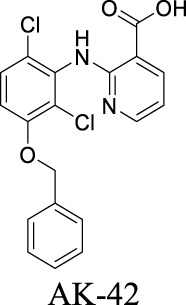	IC_50_: 17 ± 1 nM Koster et al. (2020)	Preclinical stage	2	6.78	71	389
ANO1	Ligand (Ca^2+^)-gated	Ischemic stroke; neuropathic pain	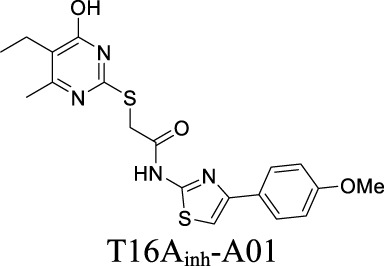	IC_50_: 0.31 ± 0.59 µM Liu et al. (2015)	Preclinical stage	2	4.83	96	416
			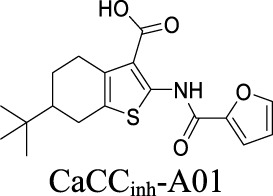	**ANO1:** IC_50_: 7.84 ± 0.62 µM; **Best 1:** IC_50_: 7.15 ± 0.65 µM Liu et al. (2015); Liu et al. (2021)	Preclinical stage	2	5.71	76	347
Best1		AD; neuron regeneration; neuropathic pain	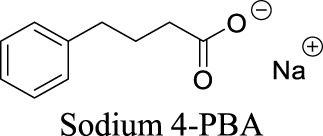	N/A	Approved drug for the treatment of urea cycle disorders	0	−100	40	186
CFTR	Ligand (cAMP)-gated	Glioma; AD; frontotemporal dementia	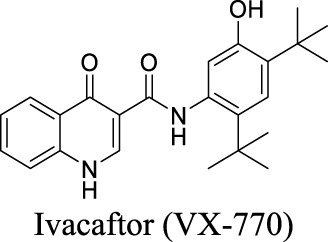	Potentiator EC_50_ G551D-CFTR: 100 nM; F508del-CFTR: 50 nM Van Goor et al. (2009)	Approved drug for CF treatment	3	3.82	78	392
			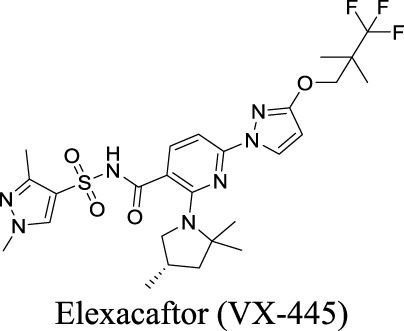	Potentiator EC_50_ G551D-CFTR: 1.12 ± 0.08 nM; F508del-CFTR: 280 nM Veit et al. (2021)	Approved as a combination drug with ivacaftor and tezacaftor for CF treatment	1	4.43	119	598
			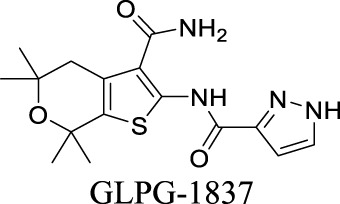	Potentiator EC_50_ G551D-CFTR: 339 nM; F508del-CFTR: 3 nM Van der Plas et al. (2018)	Phase II clinical trial	3	1.88	106	348
			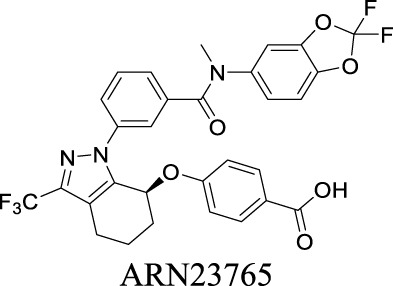	Corrector EC_50_ F508del-CFTR: 38 pM Pedemonte et al. (2020)	Preclinical stage	1	7.58	101	616
			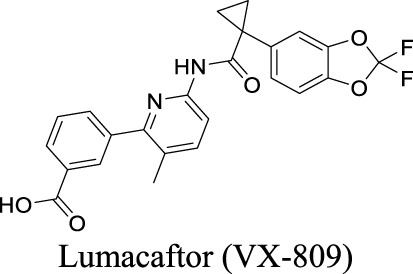	Corrector EC_50_ F508del-CFTR: 81 ± 19 nM Van Goor et al. (2011)	Approved as a combination drug with ivacaftor for CF treatment	2	6.05	97	452
			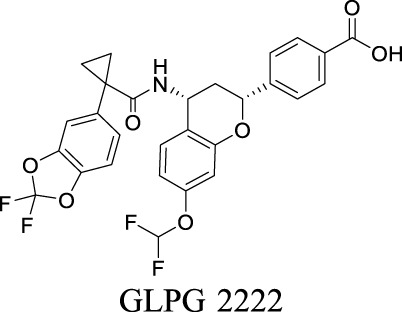	Corrector EC_50_ F508del-CFTR: 5 nM Wang et al. (2018)	Phase II clinical trial	2	6.51	103	559
VRAC	Volume-regulated	Brain injury; stroke; hyponatremia; epilepsy	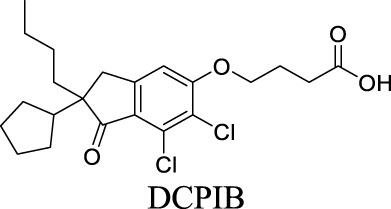	IC_50_: 4.1 µM Zhi et al. (2022)	Preclinical stage	1	7.14	64	427
			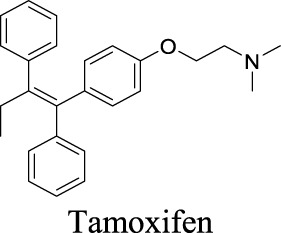	IC_50_: 4.6 µM Shen et al. (2000)	Approved drug as an estrogen modulator for breast cancer treatment.	0	6.82	12	372
			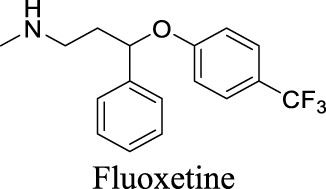	K_i_ = 6.0 ± 0.5 μM Maertens et al. (1999)	Approved drugs as selective serotonin reuptake inhibitors for antidepression	1	4.57	21	309
			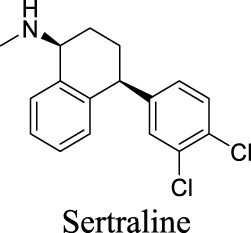	IC_50_: 2.1 ± 0.5 µM Maertens et al. (2002)		1	5.35	12	306
			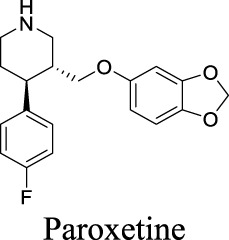	IC_50_: 2.7 ± 0.2 µM Maertens et al. (2002)		1	4.24	40	329
			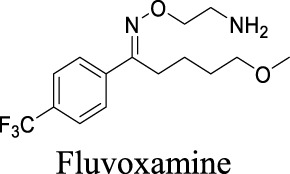	IC_50_: 12.3 ± 1.4 µM Maertens et al. (2002)		1	3.03	57	318
			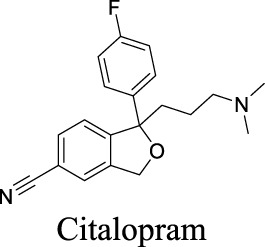	IC_50_: 27.7 ± 2.8 µM Maertens et al. (2002)		0	3.13	36	324
			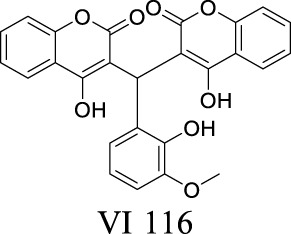	IC_50_: 1.27 ± 0.18 µM Jeon et al. (2022)	Preclinical stage	3	3.78	123	458
GABA_A_ Receptor	Ligand (GABA)-gated	Dementia; primary insomnia; epilepsy	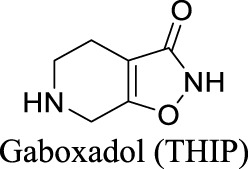	EC_50_ α4β3δ: 13 µM Hoestgaard-Jensen et al. (2014)	No longer in clinical development	2	−0.58	50	140
			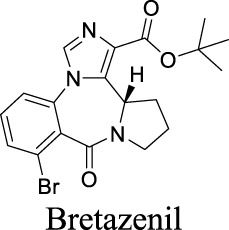	Partial agonist EC_50_ α1β1γ2: 10 nM Puia et al. (1992)	Anxiolytic drug	0	3.07	62	418
			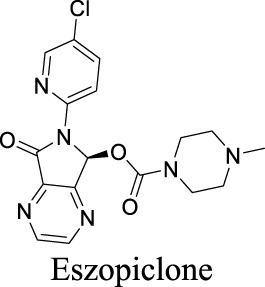	EC_50_ α1β2γ2: 301 nM; α1β2γ3: 554 nM Richter et al. (2020)	Approved drugs for the treatment of insomnia	0	1.25	90	389
			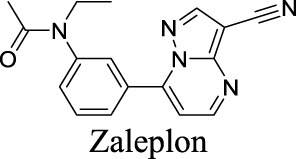	EC_50_ α1β2γ2: 203 nM; α1β2γ3: 56 nM Richter et al. (2020)		0	1.43	72	305
			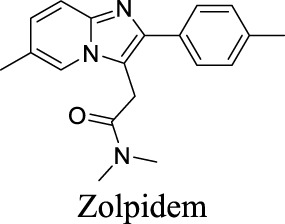	EC_50_ α1β2γ2: 230 nM Richter et al. (2020)		0	3.02	36	307
			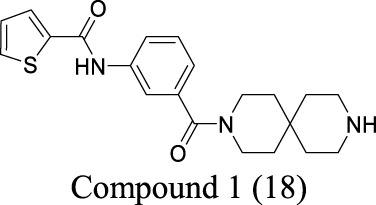	Antagonist IC_50_: α3-5 containing receptors: 37–88 nM; α1,2,6 containing receptors: 240–790 nM Falk-Petersen et al. (2020)	Preclinical	2	2.09	61	384
MAC	Solute carrier organic anion transporter family member 2A1	Cerebral edema; stroke; inflammation	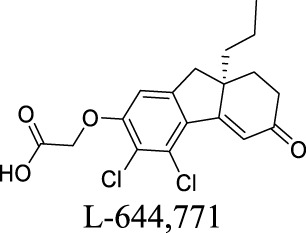	N/A	Preclinical	1	4.55	64	369

#### 2.1.2 ClC-2

In comparison to ClC-1, ClC-2 is abundantly expressed in the CNS, where it is triggered by negative membrane voltage, cellular volume change, increased intracellular Cl^−^, or extracellular acidification (Grunder et al., 1992; Jordt and Jentsch, 1997), modulating chloride efflux, neuroexcitation, myelination, and signaling transduction (Sik et al., 2000; Niemeyer et al., 2004). In hippocampal neurons, ClC-2 mediates chloride currents, a substantial part of the background conductance. The loss of ClC-2 in interneurons induces a dramatic increase of excitability, causing inhibition of principal neurons, thereby reducing overall network excitability (Rinke et al., 2010). In glia, ClC-2 has been identified as a positive modulator of oligodendrocyte maturation from precursor cells and subsequent myelin formation, repairing myeline-associated neurological disorders (Hou et al., 2018). Its function has been further demonstrated in aging study that identified the neuroprotective role of ClC-2 in the hippocampus (Cortez et al., 2010). In addition, ClC-2 is expressed in the end feet of astrocytes surrounding blood vessels, where it regulates chloride ion and blood flows (Sík et al., 2000).

Due to the wide distribution in the CNS, dysregulated ClC-2 may lead to multiple neurological disorders. ClC-2 mutation, for instance, has been suggested to be a cause of epilepsy (D'Agostino et al., 2004) although the mechanism needs further elucidation. In addition, aged ClC-2 KO mice exhibit perturbed neurotransmission patterns and increased excitation associated with astrocyte activation and neuronal degeneration (Cortez et al., 2010). Megalencephalic leukoencephalopathy with subcortical cysts (MLC) is a disease that causes seizures and developmental delay in early life, followed by a deterioration of motor functions and intellectual abilities. Pathology study identifies vacuolations in the myelin and astrocytes of MLC patients, suggesting that the disturbed ion homeostasis might be the reason for MLC development (van der Knaap et al., 1996; Duarri et al., 2011). Mutation on *GLIALCAM* is one explanation for MLC pathogenesis (López-Hernández et al., 2011). GlialCAM is a molecule that targets ClC-2 to cell junctions and increases ClC-2-mediated current, altering its functional properties (Jeworutzki et al., 2012). Aberrant GlialCAM, however, fails to target ClC-2 to cell junctions, leading to MLC disease. This observation is consistent with the animal study that shows ClC-2 KO mice develop widespread vacuolation in the white matter of the brain and spinal cord, which might be related to defective oligodendrocytes (Blanz et al., 2007).

AK-42 is a small molecule that inhibits ClC-2 with nanomolar potency (IC_50_ = 17 ± 1 nM) and rapidly and reversibly blocks ClC-2 currents. It displays unprecedented selectivity over ClC-1 and exhibits no off-target engagement against a panel of other common channels, receptors, and transporters expressed in brain tissue (Koster et al., 2020). This development provides a precise tool for future investigation on chloride-involved neurophysiology and the discovery of ClC-2-related therapeutics. In addition, peptide inhibitors have been developed as a pharmacological tool to probe ClC-2 structure/function (Thompson et al., 2009). To deliver the peptide therapeutics across BBB, brain-penetrating molecular transport vectors, such as BBB shuttle peptides, have been developed (Oller-Salvia et al., 2016). These brain-permeable peptides conjugated with therapeutics can traverse BBB through diverse mechanisms.

### 2.2 Ca^2+^-activated Cl^−^ channels (CaCCs)

Ca^2+^-activated Cl^−^ channels (CaCCs) are activated by intracellular Ca^2+^, exhibiting an outwardly rectifying current-voltage relationship at relatively low Ca^2+^ concentration while displaying a linear current-voltage relationship at higher Ca^2+^ concentration (Huang et al., 2012a).

#### 2.2.1 Anoctamins 1 and 2

Among the multiple family members in anoctamin (ANO, also known as TMEM16) channels, ANO1 and ANO2 are considered as CaCCs with defined physiological functions (Oh and Jung, 2016). In the CNS, they have been identified in the cerebellar cortex, hippocampus, and olfactory bulb, where they modulate synaptic transmissions and olfaction (Rasche et al., 2010; Huang et al., 2012b; Zhang et al., 2015). For instance, ANO1 plays a role in the network of inhibitory interneurons in the cerebellar cortex, while ANO2 may modulate the inhibitory input to Purkinje cells (Zhang et al., 2015). During CNS development, ANO1 also involves in the maturation of radial glial cells contributing to cortex development (Hong et al., 2019).

ANOs also participate in neurological diseases. For instance, ANO1 is overexpressed in various cancer cells including glioblastoma. Under this pathological condition, activation of tyrosine kinases and G protein-coupled receptors increases intracellular Ca^2+^ concentration in glioblastoma cells, triggering the gating of ANO1 (Kang et al., 2010). ANO1 also promotes cancer progression by stimulating the signaling pathway of cell proliferation (Britschgi et al., 2013; Liu et al., 2014). Suppression of ANO1 activity inhibits migration and invasion of these glioblastoma cell lines, indicating its therapeutic value (Lee et al., 2016). In addition, ANO1 elevates the excitability of dorsal-root ganglion neurons under inflammatory or neuropathic conditions, suggesting that ANO1 inhibitors can be developed as novel analgesics (Lee et al., 2014; Pineda-Farias et al., 2015). More recently, ANO2 has been identified as an autoimmune target in multiple sclerosis (Ayoglu et al., 2016).

Multiple ANO1 inhibitors have been developed and their therapeutic values have been investigated in neurological disorders. CaCCi_nh_-A01 inhibits ANO1 with IC_50_ at 7.40 µM (Table 1) (Liu et al., 2015). This inhibitor also blocks another CaCC, Best1 with similar IC_50_. In comparison, T16A_inh_-A01 partially inhibits ANO1 but has no activity on Best1 (Liu et al., 2015). Inhibition of ANO1 activity by CaCCi_nh_-A01 and T16A_inh_-A01 has been demonstrated as a tool to generate analgesia in nerve injury pain (Pineda-Farias et al., 2015). In addition, CaCCi_nh_-A01 also preserves BBB integrity, attenuates brain infract size and neurological deficits after ischemic stroke, indicating that ANO1 may become a potential target for ischemic stroke (Liu et al., 2019).

#### 2.2.2 Bestrophin1 (Best1)

Best1 is distributed in the olfactory bulb, hippocampus, and cerebellum, expressed in both neurons and astrocytes (Park et al., 2009). Best1 is activated by an increase of Ca^2+^ and induces Cl^−^ flux across cell membrane. This action leads to membrane depolarization or hyperpolarization, depending on the equilibrium potential. Best1 also plays distinct roles in the brain, where it exhibits permeabilities for several other monovalent anions, including Br^−^, I^−^, SCN^−^, HCO_3_^−^, and NO_3_^−^ (Qu and Hartzell, 2008; O'Driscoll et al., 2009). In addition, Best1 in astrocytes has been reported to regulate larger anions including GABA (Lee et al., 2010), one major inhibitory neurotransmitter, and glutamate (Park et al., 2009; Woo et al., 2012), one excitatory neurotransmitter mediated and recycled by astrocytes.

Interestingly, the expression and functions of Best1 exhibit altered patterns in astrocytes under pathological conditions. Resting astrocytes do not synthesize GABA but express Best1 at microdomains (astrocytic membrane protrusions enwrapping synaptic terminals) to regulate glutamate release targeting NMDA receptors (Oh and Lee, 2017). In Alzheimer’s disease (AD), however, astrocytes that surround Aβ plaque are activated to maintain brain homeostasis. This action triggers the synthesis of GABA in astrocytes and the redistribution of Best1 from perisynaptic microdomains to soma, from which GABA is released by astrocytes through Best1 (Oh and Lee, 2017). The GABA further diminishes the spike probability and synaptic plasticity, impacting learning and memory function (Jo et al., 2014). This evidence highlights the role of Best1 in neuron-glia crosstalk through GABA as a gliotransmitter in AD. Upregulated Best1 with associated increase of chloride currents has been observed in dorsal root ganglia after peripheral nerve axotomy and spinal nerve ligation (Boudes et al., 2009; Pineda-Farias et al., 2015). Also, Best1 KO mice exhibit decreased neurite outgrowth velocity in cultured injured sensory neurons, suggesting a positive role in regeneration (Oh and Lee, 2017).

Sodium phenylbutyrate (4-PBA) appears to act as a chaperone to improve Best1 protein folding and rescue the function of Best1 mutants, thereby improving the chloride conductance (Liu et al., 2020).

### 2.3 Cystic fibrosis transmembrane conductance regulator (CFTR)

CFTR, a cAMP-dependent ion channel, transports chloride and bicarbonate in the epithelial cells of airways, gastrointestinal and reproductive organs (Verkman and Galietta, 2021). Aberrant CFTR results in cystic fibrosis (CF) and subsequent impaired fluid and pH homeostasis, contributing to the pathology in the lungs, pancreas, livers, intestine, and testis (Verkman and Galietta, 2021). Interestingly, CNS complications occur more frequently in CF patients than other lung transplant recipients (Goldstein et al., 2000), suggesting CFTR may exist in the CNS and participate in neuronal functions. *Ex-vivo* study has identified the expression of CFTR in hypothalamus, thalamus, amygdala, and limbic system (Mulberg et al., 1995; Mulberg et al., 1998; Weyler et al., 1999), the areas regulating metabolism, food intake, sex differentiation, and energy expenditure. This distribution seems to explain the symptoms of growth failure and malnutrition in CF patients.

At the cellular level, CFTR expression has been observed in both neurons and glia (Liu et al., 2006a; Guo et al., 2009) and its expression shows different patterns depending on brain development stage (Marcorelles et al., 2014). Patients with CF show axonal dystrophy and detectable amyloid precursor protein (Goldstein et al., 2000), implying that CFTR not only performs fundamental functions in cell maturation during brain development but also contributes to neurological disorders. Decreased expression of CFTR, for instance, has been observed in the astrocytes differentiated from patients with frontotemporal dementia type 3 (Chandrasekaran et al., 2021). In AD, CFTR gene expression is downregulated in the hypothalamus, suggesting a potential role in the regulation of metabolic function during neurodegeneration (Lahousse et al., 2003). Mutation in this gene leads to exaggerated proinflammatory responses in AD (Lahousse et al., 2003). *In-vitro* study demonstrates that CFTR suppresses apoptosis of glioma cells; inhibition of CFTR function or expression suppresses the glioma cell viability, whereas overexpression of CFTR shows an opposite impact (Zhao et al., 2020). This observation is consistent with the immunohistochemistry study on the samples collected from glioblastoma patients, from which the expression level of CFTR is significantly increased (Zhao et al., 2020).

More than 2000 CFTR mutants that impact protein synthesis and stability have been identified (Veit et al., 2016). To restore the function of CFTR, two types of modulators have been developed. “Correctors” are the small molecules directly interacting with mutant CFTR to repair protein folding and improve stability. In contrast, “potentiators” correct the dysregulation by improving channel gating. For instance, ivacaftor, a CFTR potentiator, improves the chloride transport by directly binding to CFTR to mediate gating, thereby restoring protein functions (Eckford et al., 2012). Lumacaftor and tezacaftor, CFTR correctors, act as chaperones during protein folding and increase protein trafficking to the cell membrane, thereby improving protein stability (Eckford et al., 2012; Ridley and Condren, 2020). The combinations of lumacaftor/ivacaftor and tezacaftor/ivacaftor have been approved by FDA for CF treatments.

### 2.4 Volume-regulated anion channel (VRAC)

Cell volume is maintained at a dynamic equilibrium through dedicated mechanisms during transmembrane fluxes of ions and nutrients, and synthesis/degradation of macromolecules. Among multiple volume-regulatory proteins, volume-regulated anion channel (VRAC) is activated by cell swelling and transports anions including Cl^−^ along electrochemical gradients. This action leads to the efflux of water to counteract with cell swelling. In the CNS, VRAC gating triggers the release of organic osmolytes such as glutamate, impacting neuronal excitability (Kasuya and Nureki, 2022).

VRAC is a heteromeric protein and activated by cell swelling. Five isoforms named LRRC8A-E have been identified. Functional VRAC is formed by multiple LRRC8 proteins including the essential LRRC8A and at least one other family member among LRRC8B-E (Hyzinski-García et al., 2014; Voss et al., 2014). The expression ratio and different combinations of LRRC8 isoforms in this complex result in diverse properties, playing a unique role in the release of different neurotransmitters such as glutamate, aspartate, GABA, and taurine. In astrocytes, for example, the LRRC8A/D complex appears to regulate the release of uncharged osmolytes, while LRRC8A/C/D/E complex is responsible for charged molecules (Lutter et al., 2017; Schober et al., 2017).

Under pathological conditions such as traumatic brain injury, stroke, hyponatremia, and epilepsy, astrocytes swell, invading extracellular space and impacting neuronal functions (Barron et al., 1988; Manley et al., 2000; Fabene et al., 2006). These events result in the buildup of glutamate and aspartate in the extracellular space, persistently depolarizing neurons. VRACs appear to play an important role during these neurological disorders. For instance, VRACs are activated by astrocytic swelling during stroke and mediate the release of excitatory amino acids (Kimelberg, 2005) (Basarsky et al., 1999). Administration of DCPIB, a specific VRAC inhibitor, reduces infarct size in reversible middle cerebral artery occlusion and the release of glutamate in the ischemic cortical penumbra, suggesting neuroprotective effects in brain ischemia. As a fully charged anion at physiological pH, however, DCPIB is not able to traverse BBB (Zhang et al., 2008). In comparison, another VRAC inhibitor, tamoxifen, has been reported to penetrate BBB and reduce brain infarction in the stroke mouse model (Kimelberg et al., 2003). Some natural products, such as phloretin, also exhibit inhibition of VRAC and associated astrocytic amino acid release (Abdullaev et al., 2006).

### 2.5 GABA_A_-gated chloride channel (GABA_A_ receptor)

The activation of GABA_A_Rs triggers an alternation of electrochemical potential, exerting inhibitory functions to regulate neuronal excitability in the CNS. GABA_A_Rs are pentameric receptor proteins composed of at least three different proteins collected from 19 subunits, α1-6, β1-3, γ1-3, δ, ε, θ, π, ρ1-3 (Brickley and Mody, 2012). The different combination of these subunits results in varying isoforms with diverse functions (Mortensen et al., 2011; Phulera et al., 2018). The predominant synaptic GABA_A_Rs are composed of two α1-subunits, two β2-subunits, and one γ2-subunit (Zhu et al., 2018), in which γ2-subunit is a major component and drives receptor clustering at synapse (Essrich et al., 1998). In contrast, the subunit composition of extrasynaptic GABA_A_Rs has the high occurrence of α4, α5, α6, and δ subunits (Brickley and Mody, 2012). Synaptic GABA_A_Rs interact with GABA with a low affinity to generate phase conductance that inhibits postsynaptic currents in a transient and rapid manner while extrasynaptic GABA_A_Rs mediate tonic conductance in the presence of ambient GABA with a high affinity, leading to a persistent inhibition (Lee and Maguire, 2014).

Disturbances in synaptic and extrasynaptic GABA_A_Rs result in multiple neurological disorders. For instance, patients with early Parkinson’s disease (PD) develop non-motor symptoms such as sleep disturbance, olfactory loss, and gastrointestinal abnormalities, which are related to the deficits of GABAergic system (Błaszczyk, 2016). There is also a correlation between genetic alteration of GABA_A_Rs and neurodevelopmental disorders such as fragile X syndrome, Rett syndrome, and Dravet syndrome (Braat and Kooy, 2015). Mutation in extrasynaptic δ-GABA_A_Rs leads to diminished tonic inhibition and epileptic seizures (Chuang and Reddy, 2018). Recent study has also identified that antipsychotic-free patients with schizophrenia have lower extrasynaptic α5-GABA_A_Rs in the hippocampus, which is not seen in antipsychotic-treated schizophrenia patients, highlighting the potential of GABAergic modulators as therapeutic targets for schizophrenia (Marques et al., 2021).

The diverse GABA_A_R subunits create more opportunities for the development of selective modulators. As GABA_A_Rs regulate neurotransmitters, they are targets of widely-used sedative and hypnotic drugs including barbiturates and benzodiazepines, which interact with the interface between α and γ subunits of GABA_A_Rs (May et al., 2013). Ligand binding locks the GABA_A_Rs into a conformation with a better exposure to GABA to potentiate inhibitory signals (Phulera et al., 2018). The same site is targeted by inverse agonists such as β-carbolines, which have an effect opposite to that of anxiolytic benzodiazepines.

In addition, inverse agonists selective to the α5 subunit of the GABA_A_R have been reported to enhance cognition without anxiogenic and convulsant effects, highlighting the therapeutic potentials to treat memory impairment associated with AD and related dementias (Sternfeld et al., 2004). Gaboxadol is a selective agonist for GABA_A_Rs that contain δ subunits, which are mainly localized in thalamic neurons. Gaboxadol improves sleeping conditions in the Phase III clinical trial to treat primary insomnia (Wafford and Ebert, 2006). However, gaboxadol is no longer in clinical development due to limited or variable efficacy and psychiatric side effects (Roth et al., 2010).

### 2.6 Maxi anion channel (MAC)

Maxi anion channels (MACs) are highly effective electrogenic chloride-transporting systems, involved in multiple physiological events. MACs are widely expressed throughout the body and triggered by osmotic cell swelling, apoptosis, ischemia, and hypoxia (Sabirov and Okada, 2009). Comparing to other chloride channels, MACs exhibit large single-channel conductance, functioning as a highly efficient anion-transporting system, and permeability to large organic anions including pyruvate, glutamate, and ATP due to their wide pore (Jalonen, 1993; Dutta et al., 2004; Liu et al., 2008a). MACs play multiple roles in the CNS. In astrocytes, MACs regulate cell volume against swelling (Jalonen, 1993). Under ischemic or osmotic stress, MACs serve as a major ATP- and glutamate-releasing pathway in astrocytes (Liu et al., 2006b; Liu et al., 2008b), modulating glutamatergic synaptic transmission and microglia activation (Xiang et al., 2006).

Multiple studies have described small molecules that modulate MAC functions. L-644-711 blocks MAC in cultured astrocytes and regulates cell volume (Jalonen, 1993). This activity alleviates brain edema resulting from traumatic injury and hypoosmotic hyponatremia (Barron et al., 1988; Trachtman and Cragoe, 1989). Deltamethrin, a type II pyrethroid pesticide, inhibits MAC activity by decreasing open probability (Forshaw et al., 1993). In addition, classical anion-channel blockers such as NPPB, SITS, DIDS, and DPC inhibit MAC activity (Sabirov and Okada, 2009). In contrast, tamoxifen, a VRAC blocker used to treat breast cancer, activates MAC (Sabirov and Okada, 2005). Ivermectin and pentobarbitone significantly activate MAC, providing a rationale for effective therapy against pyrethroid-induced neurotoxicity (Forshaw et al., 2000).

## 3 Discussion

**FIGURE 1:**
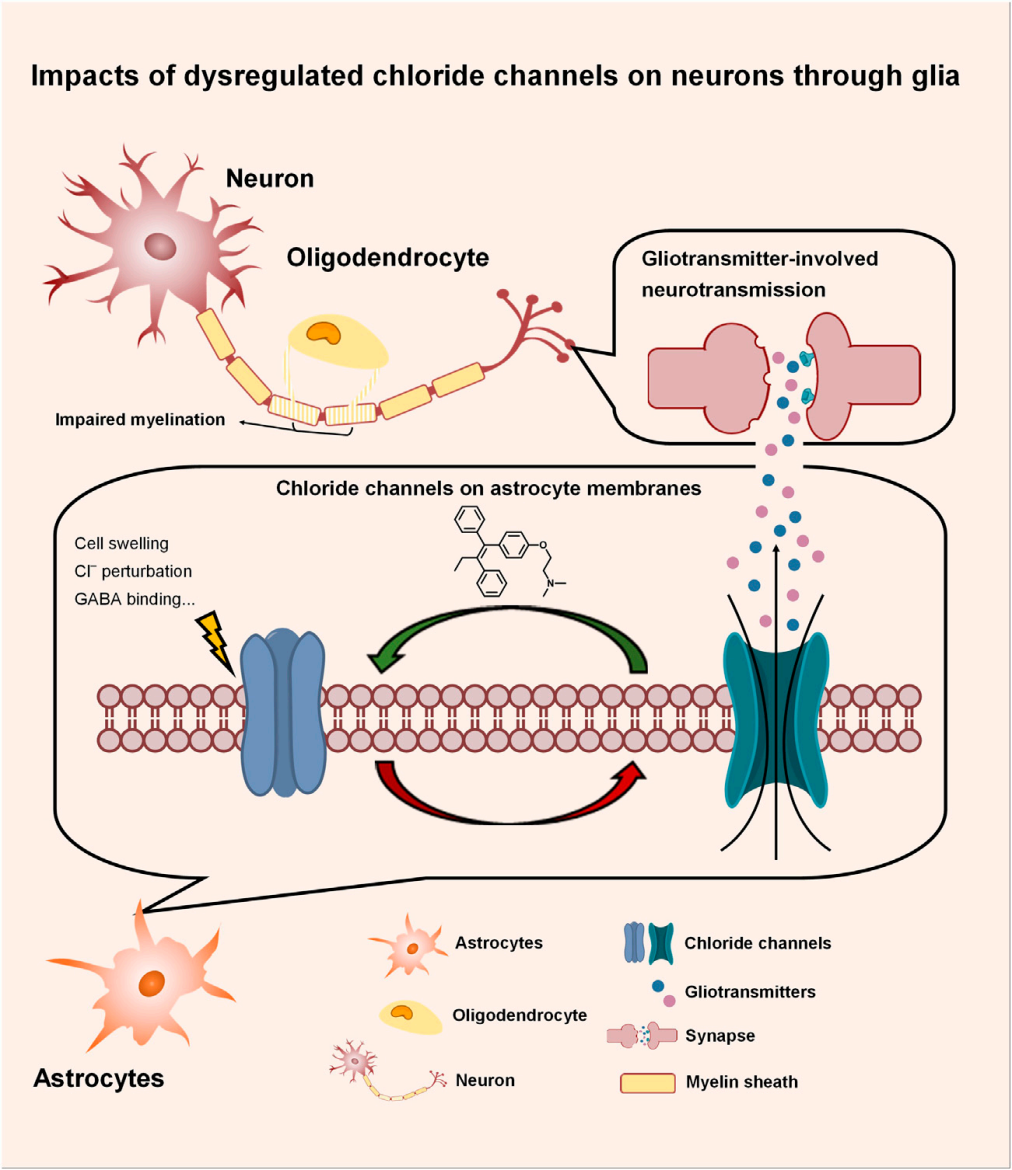
Impacts of dysregulated chloride channels on neurons through glia. Chloride channels in the CNS not only directly modulate neuronal excitability, but also indirectly impact neuronal functions through the gliotransmitters released from astrocytes *via* gating organic anions such as glutamate and GABA. In neurological disorders, dysregulated chloride channels release excessive neurotransmitters, causing neuronal impairment.

Chloride channels in the CNS not only directly modulate neuronal excitability, but also indirectly impact neuronal functions through the gliotransmitters released from astrocytes *via* gating organic anions such as glutamate and GABA, as depicted in Figure 1. In neurological disorders, dysregulated chloride channels release excessive neurotransmitters, causing neuronal impairment. Our literature survey highlights astrocytic swelling that impacts brain function. This section further discusses the impacts of dysregulated chloride channels during astrocytic swelling and elucidates their contributions to the pathogenesis of neurological disorders. Along with a summary of the small molecules that modulate chloride channels, we also propose the potential strategy of optimizing exiting drugs to brain-penetrants, supporting future CNS drug discovery.

Astrocytes participate in fundamental roles in the CNS, where they maintain ion homeostasis, provide essential nutrients, and mediate neuronal excitability through gliotransmitters such as glutamate, GABA, and ATP. In physiological conditions, astrocytes release gliotransmitters to the network with neurons upon receptor activation (Gordon et al., 2005), osmotic perturbation (Darby et al., 2003), and deprivation of extracellular Ca^2+^ (Suadicani et al., 2006). During neurological disorders such as ischemia, however, ion-transporting systems of astrocytes and BBB endothelial cells are dysregulated, contributing to astrocytic swelling and vasogenic edema. Astrocytes are the major cell type that swells in gray matter (Kimelberg, 2000). As the most abundant cells in the CNS, astrocyte swelling significantly invades extracellular space, elevating intracranial pressure, reducing blood flow, and subsequently leading to tissue damage. As the CNS is encased within a rigid skull unlike other tissues, edema in the brain is life-threatening (Lafrenaye and Simard, 2019). In addition to these direct impacts, astrocytic swelling also dysregulates its own cellular function and induces secondary neurotoxicity. Astrocytes initiate cellular machinery against swelling to re-establish their pre-swelling volume by losing intracellular ions and excitatory amino acids such as glutamate, which induces excitotoxicity and subsequent neuronal injury (Kimelberg, 2000). As discussed in the last section, multiple chloride channels such as VRAC and Best1 have been suggested to modulate the release of excitatory amino acids, highlighting their roles in neuronal function impairments during neurological disorders and the therapeutic value of chloride-channel modulators.

In addition to the regulations of membrane potential and gating anions, chloride channels also participate in cell apoptosis through endoplasmic reticulum (ER) stress. Ischemia reperfusion injury and aging generate reactive oxygen species (Octavia et al., 2012; Liochev, 2013), which in turn activate VRACs, further inducing ER stress and downstream apoptosis (Shen et al., 2014). The role of ER stress in the pathogenesis of neurological disorders including AD, PD, and amyotrophic lateral sclerosis, is well documented (Lindholm et al., 2006). Treatment of chloride-channel blockers appears to prevent apoptosis through ER-stress pathway, reinforcing their therapeutic values against neurodegenerative diseases (Shen et al., 2014).

Designing molecules to traverse the BBB is a challenging hurdle in CNS drug discovery. The BBB is a layer that prevents hydrophilic substances, charged molecules, and proteins from entering into the extracellular fluid of the CNS from the circulating blood to protect brain tissues from pathogens and other neurotoxins. The BBB exchanges brain-necessary substances such as glucose, amino acids, and ions through selective and active transporting systems. Certain small molecules may also diffuse passively through the BBB and enter the brain. However, the BBB has a dedicated efflux system composed of breast cancer resistance protein (BCRP) and multiple drug resistance 1 (MDR1), restricting their substrates from the CNS.

In past decades, tremendous efforts have been made to regulate chloride homeostasis. Although most drugs were developed to target the peripheral systems, they provide a valuable reference for the drug discovery/repurpose towards CNS diseases. To support structure optimization for future endeavors, we summarized the small molecules that modulate chloride channels with appealing activity and specificity in Table 1 and calculated their physicochemical properties to identify the property to be optimized. cLogP stands for calculated logarithm of partition coefficient P. It is the ratio of compound concentration in a mixture of two immiscible solvents (such as water and *n*-octanol) at equilibrium, evaluating how hydrophobic a compound is. Hydrogen bond donors (HBDs) are the electronegative atoms, such as O or N, covalently bonded to hydrogens that can be donated. HBDs provide opportunities to molecular recognition, structural stability, drug partition, and permeability (Coimbra JTSFeghali et al., 2021). However, these polar moieties decrease the affinity toward the hydrophobic membrane and increase the energy penalty required to desolvate the molecule from water (Alex et al., 2011). Consequently, HBD is considered as one important parameter in medicinal chemistry. Polar surface area (PSA) is the surface area of all polar atoms in one molecule. CNS drugs usually have a relatively low PSA value than non-CNS drugs. These parameters have been integrated in multiple algorithms to predict compound permeability to BBB (Wager et al., 2010; Gupta et al., 2019). The preferred ranges of these parameters are as followed: 2 < cLogP < 4, HBD < 3, PSA < 90, and molecular weight (MW) < 450 (Wager et al., 2010).

By analyzing the structures shown in Table 1, we noticed that some molecules are built with the moieties that are not favorable to CNS penetration, such as carboxylate and primary sulfonamide. Several strategies have been proposed for structural modification of small molecules to improve BBB penetration: increasing lipophilicity, reducing hydrogen bond donor capacity, reducing PSA, enhancing rigidity, and reducing pKa (Xiong et al., 2021). Herein, we discuss the possible structure optimization of selected chloride channel mediators from Table 1 as case study and evaluate these modifications using physicochemical parameters.

**SCHEME 1:**
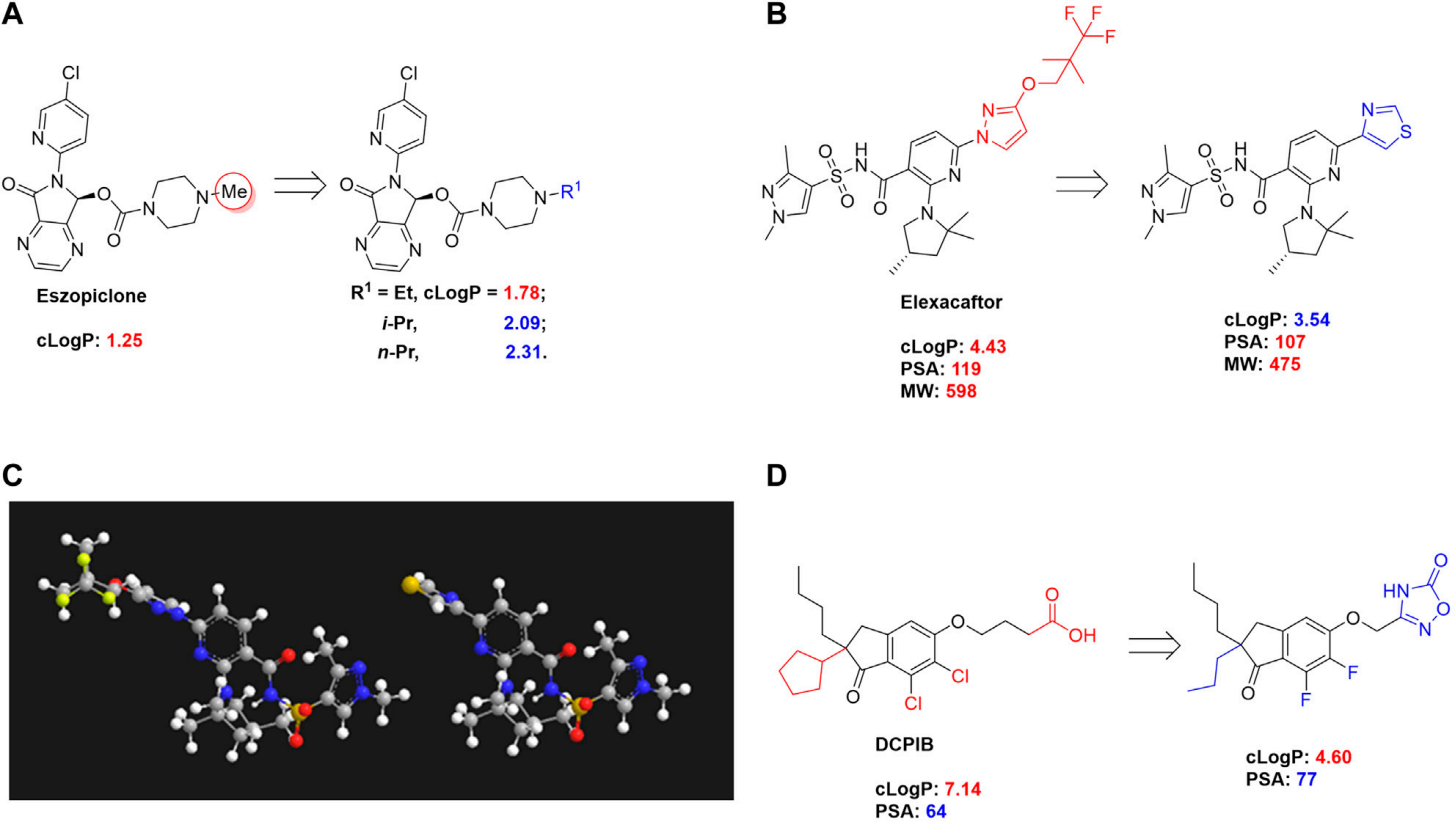
Examples of structure optimization to improve physicochemical properties. **(A)** Eszopiclone: methyl group (highlighted in red cycle) is replaced with a different group represented by R^1^. cLogP value is improved with substitution by ethyl or propyl groups. **(B)** Elexacaftor: this design replaces the pyrazole in the middle with a thiazole ring to retain the molecule orientation to the target. **(C)** Molecule orientation before (left) and after (right) structure modification for elexacaftor. **(D)** DCPIB: bio-isosteric replacement of carboxylic acid with oxadiazolone.

Eszopiclone has one methyl group on the piperazine ring as highlighted in Scheme 1A, which is not directly involved in binding interactions with its target based on previous docking studies (Hanson et al., 2008). Substitution of a methyl group with other aliphatic chains such as an ethyl or propyl group improves cLogP value from 1.25 to 1.78 or 2.31, respectively. In contrast, elexacaftor in Scheme 1B shows high cLogP, PSA, and MW. The binding study has demonstrated that the sulfonamide and amide groups of elexacaftor form hydrogen bonds with CFTR. Elexacaftor also interacts with transmembrane helix through electrostatic and van der Waals interactions (Fiedorczuk and Chen, 2022), suggesting that all the moieties of elexacaftor orientate into one docking position and contribute to binding interactions. In this case, we propose a design to replace the pyrazole ring in the middle with a thiazole ring, in which the heteroatoms may serve as hydrogen bond accepters. In addition, the aromatic system of thiazole may also retain this moiety into a planer shape and provide hydrophobicity contributing to binding interaction with CFTR. To determine whether the molecule retains the comparable pose to fit in the binding pocket, we compare the 3D structures of elexacaftor before and after the modification. As shown in Scheme 1C, the molecules superimpose from the pyridine moiety to the pyrazole ring, suggesting the modified compound can fit in the same binding pocket with a similar binding pattern as elexacaftor. In addition to direct modifications of molecule structure, bio-isosteric replacement also can create a new molecule with similar biological properties to the parent compound but with optimized bioavailability, which has been commonly adopted in medicinal chemistry. For instance, carboxylate imparts significant polarity, thereby strongly impacting pharmacokinetics and drug distribution across BBB. Carboxylic acid may also undergo glucuronidation during phase II metabolism facilitating renal clearance, possibly causing another issue for CNS-drug design. We thereby devise an optimization of the carboxylic acid moiety in DCPIB to its bio-isostere oxadiazolone in Scheme 1D. The cLogP value is then optimized from 7.14 to 4.60.

The rationale of proposed structure optimizations in the case studies above is solely based on the calculated physicochemical properties and previous binding studies. Minor changes on compound structure could change molecule’s orientation and thereby significantly impact compound activity. Thus, *in vitro* as well as *in vivo* assessments would be required to validate compound potency after structure modifications.

## Abbreviation

4-PBA, phenylbutyrate; Aβ, beta-amyloid; AD, Alzheimer’s disease; ANO, anoctamin; BBB, blood-brain-barrier; BCRP, breast cancer resistance protein; Best1, bestrophin 1; CaCC, Ca^2+^-gated chloride channel; CF, cystic fibrosis; CFTR, cystic fibrosis transmembrane conductance regulator; cLogP, calculated logarithm of partition coefficient P; CNS, central nervous system; ClC, voltage-gated chloride channel; ER, endoplasmic reticulum; FDA, food and drug administration; GABA, γ-aminobutyric acid; HBA, hydrogen bond acceptor; HBD, hydrogen bond Donor; HD, Huntington disease; MAC, maxi anion channel; MDR1, multiple drug resistance 1; MLC, megalencephalic leukoencephalopathy with subcortical cysts; MW, molecular weight; PD, Parkinson’s disease; THIP, 4,5,6,7-tetrahydroisoxazolopyridin-3-ol; TPSA, topological polar surface area; VRAC, volume regulated anion channel.
